# Sleep, Function, Behaviour and Cognition in a Cohort of Children with Down Syndrome

**DOI:** 10.3390/brainsci11101317

**Published:** 2021-10-04

**Authors:** Jasneek K. Chawla, Anne Bernard, Helen Heussler, Scott Burgess

**Affiliations:** 1 Department of Paediatric Respiratory & Sleep Medicine, Queensland Children’s Hospital, Brisbane, QLD 4101, Australia; h.heussler@health.qld.gov.au (H.H.); scott@qclass.com.au (S.B.); 2 School of Clinical Medicine, University of Queensland, Brisbane, QLD 4072, Australia; 3 QFAB Bioinformatics, Institute for Molecular Bioscience, University of Queensland, Brisbane, QLD 4072, Australia; a.bernard@qfab.org

**Keywords:** sleep, Down syndrome, paediatric, outcomes

## Abstract

Objective: To describe the sleep problems experienced by children with Down syndrome attending a tertiary sleep clinic and relationship with behaviour, function and cognition. Methods: Data were collected from children with Down syndrome aged 3–18 years old. Carers completed the Abbreviated Child Sleep Habits Questionnaire, Child Behaviour Checklist and Life-Habits Questionnaire at enrolment. Cognitive assessment (Stanford-Binet 5) was undertaken by a trained psychologist. Children received management for their sleep problem as clinically indicated. Results: Forty-two subjects with a median age of 6.8 years (Interquartile Range-IQR 4.5, 9.8) were enrolled. A total of 92% were referred with snoring or symptoms of Obstructive Sleep Apnoea (OSA), with 79% of those referred having had previous ENT surgery. Thus, 85% of all participants underwent a sleep study and 61% were diagnosed with OSA (OAHI ≥ 1/h). Based on questionnaires, 86% of respondents indicated that their child had a significant sleep disorder and non-respiratory sleep problems were common. Non-respiratory problems included: trouble going to sleep independently (45%), restless sleep (76%), night-time waking (24%) and bedtime resistance (22%). No significant correlations were found between sleep measures (behavioural and medical sleep problems) and the behavioural, functional or cognitive parameters. Conclusion: Sleep disorders were very common, especially non-respiratory sleep problems. OSA was common despite previous surgery. No association was found between sleep-related problems (snoring, sleep-study-confirmed OSA or non-respiratory sleep problem) and parent-reported behavioural problems, functional impairments or intellectual performance. This may reflect limitations of the measures used in this study, that in this population ongoing problems with daytime function are not sleep related or that a cross-sectional assessment does not adequately take into account the impacts of past disease/treatments. Further research is required to further evaluate the tools used to evaluate sleep disorders, the impact of those disorder on children with Down syndrome and interventions which improve both sleep and daytime function.

## 1. Introduction

Down syndrome (DS) or Trisomy 21 has an estimated prevalence between 1/650 to 1/1000 live births [1,2,3] and is the commonest genetic cause of significant intellectual disability [4]. Numerous factors contribute to the highly variable impairment seen in individuals with DS, including genetics, epigenetics, early neural development and environmental factors [5]. Sleep problems have been hypothesised to contribute to this unpredictable phenotype and may represent a modifiable target.

Several clinical features of DS lead to disturbed sleep and/or increased risk for sleep disordered breathing (SDB) [4]. Obstructive sleep apnoea (OSA) results from hypotonia, macroglossia and midface hypoplasia. Associated co-morbidities, such as obesity and congenital heart disease, may also contribute to an increased risk of disrupted sleep or abnormal breathing in sleep [4].

Polysomnography (PSG) studies undertaken in children with DS suggest an elevated prevalence of OSA, ranging between 31–79% [6,7,8,9,10], compared to 1–5% prevalence in the general paediatric population [11]. Non-respiratory sleep difficulties such as bedtime resistance, sleep anxiety, night waking, parasomnias and daytime sleepiness have all been reported commonly in children with DS [12,13,14,15,16]. Studies involving both community and referred cohorts of children with DS demonstrate a high prevalence of these non-respiratory sleep issues [12,17].

The negative effects of poor sleep on behaviour, function and cognition in typically developing children are well documented [18,19,20,21,22,23]. A small number of studies in children with DS have demonstrated poorer outcomes in those with co-existing sleep problems, compared to those without reported sleep difficulties [24,25,26,27,28,29]; Breslin et al. found that school-age children with DS and OSA had poorer executive function than those without OSA [24]. Similarly, Chen et al. demonstrated that features of OSA were significantly associated with poor verbal fluency and attention in adolescents and young adults with DS [26]. Edgin et al. found that in pre-school children with DS, poor sleepers showed specific difficulties with expressive language compared to good sleepers [27]. Studies of behaviour have had mixed results, possibly due to methodological differences, but two large studies have shown a positive relationship between sleep and poor behaviour in this population [29,30]. In the only study to assess functional ability, Churchill et al. found that sleep problems were negatively associated with accomplishment of daily activities in children with DS [25]. To date, no studies have evaluated the impact of treating sleep disorders on these outcomes in children with DS.

The aims of this paper were to: 1. Evaluate the rates of medical and non-medical sleep problems in a cohort of children with Down syndrome attending a tertiary sleep clinic; 2. Assess the degree and nature of behavioural problems, functional and intellectual impairment; 3. Explore the association between sleep disorders and behavioural problems, functional limitation and cognitive measures to gain insight into how improvements in sleep might benefit children with DS. We hypothesise that the proportion of both OSA and non-medical sleep problems will be high in this cohort of children and anticipate that children with sleep problems may have worse behaviour, function and cognition.

## 2. Materials and Methods

Data were collected from children with DS aged 3–16 years of age attending a tertiary sleep medicine clinic over a 12-month period from April 2017. At enrolment, families completed questionnaires regarding sleep, behaviour and function. A psychologist performed cognitive testing. Comorbidities, past medical history and sleep assessments (including polysomnography) were obtained from electronic medical records. The Children’s Hospital Queensland and University of Queensland Research Ethics Boards approved this study (HREC/16/QRCH/312/AM01).

### 2.1. Exclusion Criteria

Children without DS and those with DS <3 yrs of age were excluded. Those under 3 years of age were not included due to the difficulties in consistently assessing function and development at this age. 

### 2.2. Outcome Measures

#### 2.2.1. Child Sleep Habits Questionnaire

Primary carers of recruited children completed the Child Sleep Habits Questionnaire-Abbreviated CSHQ-A [31]. The CSHQ is the most commonly used tool for assessing sleep problems in typically developing children [32] and has been previously applied by other groups in children with DS [12,13,16,17,33,34]. The CSHQ-A is an adapted version, which is half the length (22 questions) and at the time of commencement of this study was the only abbreviated version available. Although this is a non-standard version of the CSHQ, the CSHQ-A was chosen primarily because its length is half that of the original version of the CSHQ. This was a key consideration for our participants, who were completing multiple detailed questionnaires, and this therefore reduced the questionnaire burden for parents in this group. Despite no formal validation studies, this version has been successfully used in studies of children with developmental disabilities including children with Autism Spectrum Disorder (ASD) [35], Attention Deficit Hyperactivity Disorder (ADHD) [36] and Duchenne Muscular Dystrophy (DMD) [37]. Items are rated on a five-point scale (score 1–5) ranging from “always” if the sleep behaviour occurred 7 times in the past week, “usually” if the behaviour occurred 5–6 times in the past week, “sometimes” for 2–4 times that week, “rarely” for 1 time that week and “never” for 0 times that week. Five questions (Q1, 2, 3, 10 and 19) are reverse scored, with 1 indicating “always” and 5 indicating “never”. The cut-off for clinically relevant sleep problems used in other studies with the CSHQ-A was 41 in one study and 30 in another [35,36]. In the current study, a total score of more than 41 was taken as abnormal and indicative of sleep problems [31,32].

#### 2.2.2. Life Habits Questionnaire

The Life Habits Questionnaire (Life-H) measures functional ability. The Life-H for children is a validated measure to assess the social participation of children with disabilities, regardless of the type of underlying impairment [38].

#### 2.2.3. Child Behaviour Checklist

The CBCL is a widely used method of identifying emotional and behavioural problems in children [39,40], including those with developmental delay [41,42,43], and has been previously utilised in studies involving children with DS [44,45].

#### 2.2.4. Stanford–Binet Intelligence Scales-5th Edition

Where possible, the Stanford–Binet Intelligence Scales 5th Edition (SB-5) developmental assessment was undertaken by a trained psychologist. This is a validated tool for evaluation of cognitive ability in children aged >2 yrs [46] and has been used in children with DS previously [47,48]. This cognitive test was chosen both due to its previous use in children with DS and because it consists of a specific non-verbal component. This is particularly important in this population, due to the profound deficits in language development that are seen in this group [49].

#### 2.2.5. Polysomnography Data

If a participant underwent polysomnography (PSG) clinically, this information was obtained from their electronic medical records. Full overnight PSGs were performed and scored manually in accordance with American Academy of Sleep Medicine (AASM) guidelines [50] using Remlogic data acquisition and analysis systems. Sleep state was determined by electroencephalography (using AASM [50] recommended EEG derivations based on the international 10–20 system for EEG electrode placement 8-lead set-up), electrooculography and submental electromyography. Respiratory status was evaluated using pulse oximetry, oronasal airflow measurement using nasal pressure transducer and thermistor, chest and abdominal movements with respiratory inductance plethysmography and carbon dioxide measured using transcutaneous CO_2_ (TcCO_2_). Cardiac rhythm was monitored by electrocardiography. Audio and visual recordings were also used in the data analysis. All studies were interpreted for clinical purposes by experienced paediatric sleep physicians.

### 2.3. Statisical Analysis

Descriptive statistics were used to describe the cohort with participants’ demographic information and polysomnographic data (where available). Mean and standard deviation (SD) was used for normally distributed continuous data or median and inter-quartile range (IQR) when normality was not met. Normality was assessed using the Shapiro–Wilk test. Categorical variables were presented using frequencies and percentages. The overall relationship between sleep problems and demographic, functional, behavioural and developmental outcomes was assessed using a Pearson’s correlation coefficient or Spearman rank coefficient (when appropriate). For children who underwent PSG, a Mann–Whitney U Test was performed to determine any difference between sleep, functional, behavioural and developmental outcomes in children with confirmed OSA (OAHI ≥ 1/h) and those without (OAHI < 1/h). All analyses were performed using the R statistical software [51].

## 3. Results

Data were available for 42 participants (male = 20) with a median age of 6.8 years (IQR 4.5, 9.8). Table 1 summarises demographic information.

### 3.1. Sleep Treatments Prior to Recruitment

Table 1 summarises the procedures children had received prior to recruitment. In terms of other management, four participants (10%) had used home oxygen therapy prior to recruitment and one was still on therapy. Eight participants (19%) had commenced CPAP therapy prior to recruitment and were continuing therapy. Six participants (14%) had been prescribed melatonin for sleep onset and maintenance issues but only one was still taking melatonin regularly.

### 3.2. Sleep Data

CSHQ data were available for all 42 participants and 36 (86%) had a sleep study during the period of the study (Table 2). A total of 86% of children had a total CSHQ score >41, indicating sleep problems, and two had a score of 41 exactly. The median overall CSHQ score was 52.5 (IQR 46.5, 62.5). Adjusting for the number of questions in each sub-score, the highest scores were in sleep disordered breathing, sleep anxiety and parasomnias. The sleep disordered breathing scale is only based on one question, which asks about whether the child snores loudly. A total of 78% of families answered “always”, “usually” or “sometimes” for this question. This would be consistent with 91.9% of children in this cohort being referred to the clinic for evaluation of snoring or symptoms of OSA. Sleep anxiety refers to the ability to sleep independently and in the dark and the parasomnias sub-score includes questions regarding movement and restlessness during sleep, teeth grinding and waking screaming/inconsolably. 

Ten questions had more than 20% of respondents achieve a score of >3 for the individual question, meaning that they answered “always” or “usually” (Table 2). This included 76.2% reporting restlessness and moving a lot during sleep as a feature of their child’s sleep and 47.67% identifying that their child falls asleep while involved in activities, suggesting daytime sleepiness. Interestingly, just under half of the participants (45.2%) were falling asleep alone in their bedroom most of the time, but the same proportion (45.2%) were also reported to require a parent in the room to fall asleep. Early morning waking was a commonly reported feature (40.4%). Day naps were reported to occur “always” or “usually” in 28.5% of participants but on further evaluation this appeared to be age-related, with the children with the higher scores being under 5 years of age. 

A total of 61% (n = 22) of participants had OSA using a definition of Obstructive Apnoea-Hypopnoea Index (OAHI) of ≥1/h with a median apnoea-hypopnea index/hour (OAHI) of 5.2/h (IQR 1.9, 11.1). The severity of obstruction was mild (28%; OAHI 1–5/h), moderate (14%; OAHI 5–10/h) and severe (19%; OAHI > 10/h), with two outliers with very severe disease who had an OAHI of 106.4/h and 129.9/h, respectively. The degree of obstructive sleep apnoea did not vary with age, although both the central apnoea index (CAI) and arousal index (AI) decreased with age, as expected.

### 3.3. Functional Assessment

Life-H data were obtained for forty participants, with two participants completing the form incorrectly (Table 3). Median total Life-H score was 4.4 (IQR 3.6, 5.6), reflecting reduced overall social participation and function. Participants scored highest in the “interpersonal relationships” sub-score, which asks about the child’s relationships with other family members and general social interactions. Lowest scores were in “communication” and “responsibilities”.

### 3.4. Behavioural Assessment

CBCL assessment was available for all forty-two participants (Table 4). The median Total T-score was 57.0 (IQR 51.8, 64.0), with 26% (*n* = 11) of participants scoring in the clinical range of concerns and 10% (*n* = 4) in the borderline clinical range. Scores for internalising and externalising problems were similar, with approximately one third of participants being in the clinical or borderline clinical concern range for each sub-domain. Children scored highest in social, thought and attention problem sub-domains, with attention problems being the most clinically significant of these. 

### 3.5. Psychology Assessment

A total of 30 of the 42 (71%) participants underwent SB-5 testing (Table 5). In the other 12 participants, assessment was either not possible to coordinate (*n* = 7), parents declined (*n* = 1) or participants were unable to undertake the test when attempted (*n* = 4). A total of 63% of participants had moderate impairment and 27% mild impairment based on Full-Scale IQ scores (FSIQ). Only 3 children (10%) had an FSIQ within the normal range and no children had severe impairment. Non-verbal IQ was higher than verbal IQ with 23% of participants scoring within normal range for non-verbal IQ compared to only 3% (1 participant) for verbal IQ. 

### 3.6. Relationship between Sleep and Outcomes at Baseline

**Figure 1:**
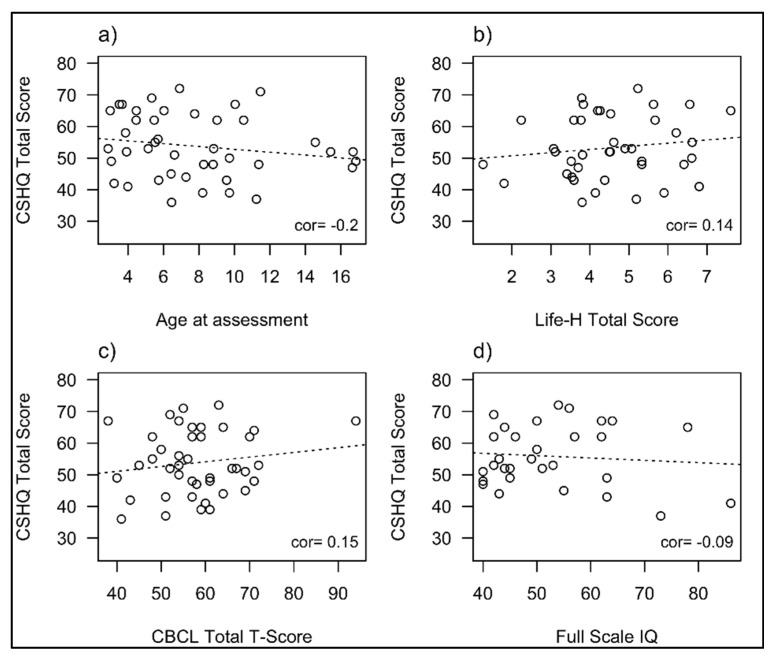
Relationship between sleep behaviour and age (**a**), functional ability (**b**), general behaviour (**c**) and IQ (**d**).

**Figure 2:**
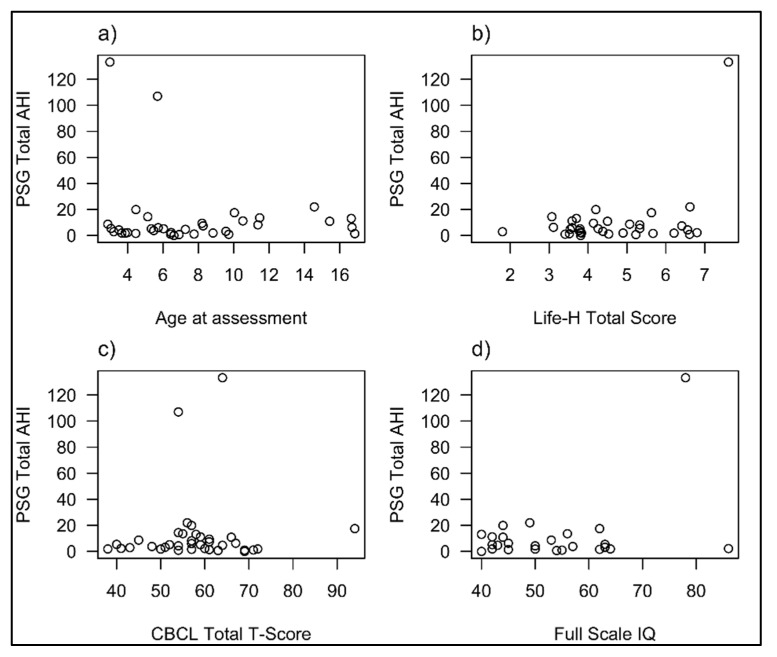
Relationship between sleep disordered breathing and age (**a**), functional ability (**b**), general behaviour (**c**) and IQ (**d**).

Correlations between a range of sleep study measures and variables from the CSHQ and behavioural, functional and IQ measures were sought. This included evaluating the relationship between PSG parameters (sleep efficiency, AHI, OAHI, CAI, CO_2_ Max and Arousal Index) and CSHQ scores and sub-scales, against age, BMI, Life H total score, CBCL total T-scores, Internalising and externalizing T-scores and SB-5 NVIQ, VIQ and FSIQ. No statistically significant correlations were seen between any sleep variables with the test variables. Figure 1 displays the relationship between CSHQ total scores and age (a), overall functional ability (b), general behaviour (c) and IQ (d) and Figure 2 displays the relationship between total AHI (Apnoea-Hypopnea Index) on PSG and these variables in those participants who had a sleep study. The two children with very severe OSA represent the outliers on these graphs.

**Figure 3:**
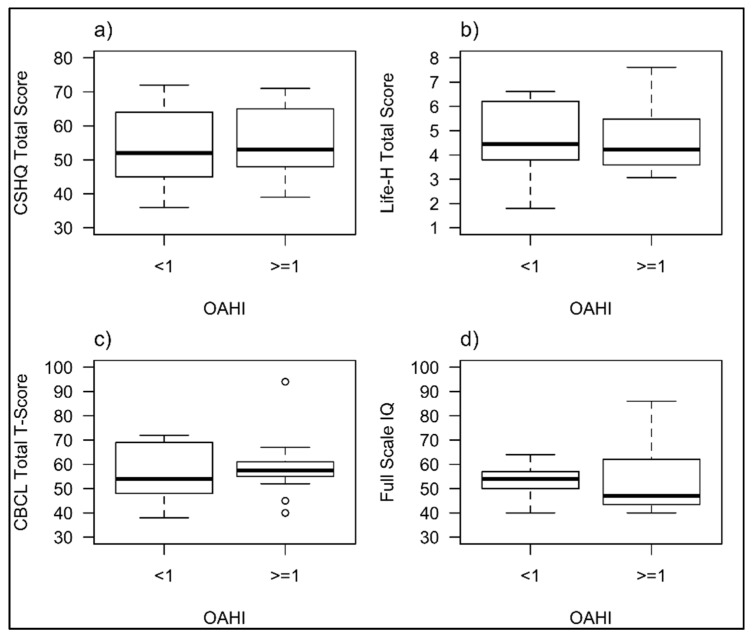
Comparison of CSHQ scores (**a**), functional ability (**b**) general behaviour (**c**) and IQ (**d**) between children with and without OSA on PSG.

A sub-group analysis was undertaken in those children who underwent PSG (*n* = 36), grouping children by OAHI into those with OSA (OAHI > 1/h) and those without (OAHI ≤ 1/h). A Mann–Whitney test was undertaken to compare participants and demonstrated no significant difference in sleep behaviour (U = 161, *p* = 0.835), functional ability (U = 131, *p* = 0.769), general behaviour (U = 175, *p* = 0.511) and IQ scores (U = 65, *p* = 0.718) between children with OSA and those without (Figure 3).

## 4. Discussion

This study describes the characteristics of a cohort of children with DS attending a tertiary sleep medicine clinic and evaluates the potential relationship between sleep and day to day function. As expected, the proportion of sleep problems in this cohort was high. Children were primarily referred for assessment of sleep disordered breathing (SDB). A total of 61% of participants who underwent a PSG were diagnosed with OSA (OAHI ≥ 1/h) despite 79% having already had one and 26% more than one previous ENT procedure. This indicates a high degree of residual OSA in children with DS who require additional treatment after ENT surgery. CPAP is often tried in this situation, but there are limited data regarding the success of sustaining therapy in this cohort. Behavioural problems commonly co-exist in this population and may contribute to difficulties with CPAP adherence.

A total of 86% of participants had a CSHQ score that was indicative of a sleep problem. Restlessness and moving a lot during sleep was a particularly common feature (76%). Restlessness did not appear to be related to a diagnosis of OSA and appears to be an independent problem. Sleep anxiety was common irrespective of age, with 45% of children regularly (always or usually) requiring a parent to be present in the room when falling to asleep. However, the same proportion were also reported to be able to regularly fall asleep alone in their own bed. This suggests that this is a skill that might be learnt and additional family and environmental factors may contribute to this difference between participants. For clinicians, this highlights that sleep associations will be important to discuss with families and providing strategies to target this area may assist families who are struggling.

Night wakings once or more than once a night was reported “always” or “usually” by 43% of respondents regardless of age, indicating that a significant proportion of children with DS have regular disturbed night-time sleep. This is further supported by 47.6% of families reporting that their child routinely (always or usually) falls asleep while involved in activities. These two findings suggest that night-time sleep disruption may contribute to poor daytime function. 

Sleep physiology and sleep–wake patterns may differ in children with DS, resulting in them struggling to learn how and when to fall asleep. This is supported by mouse models of Down syndrome, which have consistently shown sleep- and rhythm-related disturbances. However further study in human subjects is still required [52]. Psychological parental factors likely impact on the ability to achieve a consistent, disciplined approach to their child’s sleep pattern and to instill independent sleep habits [53]. Measures used in TD children, such as parental education to encourage healthy sleep habits, behavioural interventions and selective use of pharmacological treatments, can be used in children with DS but success is likely to be dependent on parental capabilities and commitment, as well as the child’s willingness and ability to comply [54]. The use of behavioural interventions and standardised programs has not been well described specifically in children with DS, with a lack of studies in this area [55]. This study supports the need for further work in this area to guide optimal clinical management. The relative strength of children in terms of non-verbal skills suggests that interventions should have a non-verbal element.

Behavioural assessment in our cohort demonstrated similar results to that of Esbensen et al. [56] Their study of 88 school-age children with DS found 33.3% scored in the range of clinical concern for “total problem score” compared to 26% in our group. The same three sub-domains of social, thought and attention problems were highest in both groups and the least problems were found in the anxious/depressed sub-domain. We did not find a correlation between either a diagnosis of sleep behavioural problems or OSA and general behavioural problems. However, even large-scale, well-designed randomised controlled trials, such as the CHAT [23] and POSTA [57] studies, have only demonstrated modest improvements in behaviour after treatment of OSA with adenotonsillectomy, suggesting that the relationship is more complex and that behavioural difficulties have a multifactorial aetiology. It is also possible that existing behavioural measures do not address the specific areas of behaviour that are affected by sleep difficulties in this cohort of children.

A direct relationship between either sleep behavioural problems or OSA and cognition was not seen. This could be explained by a multifactorial aetiology for the cognitive difficulties in DS or the inability of the tests used to evaluate OSA and/or cognition.

This study has several recognised limitations; although larger than many, the sample size is still small, therefore making generalisation of findings difficult. The measures used in this study are largely subjective, being questionnaire based. The addition of sleep study data has enabled quantification of SDB objectively. Actigraphy was also attempted. However, most children were not able to consistently tolerate wearing the device.

It is acknowledged that this is a population of children referred for sleep problems and most children had already received some treatment for sleep problems prior to recruitment. Whilst the primary aim of this study was not to perform a comparative study, we recognise that our data would be more clinically meaningful if placed in context to that from other groups of children. Subsequent to this study we have recruited a community-based sample of children with DS and demonstrated that the prevalence of sleep disturbances in this community sample was very similar to that seen in the referred tertiary population of children with DS in this current study (91% in community group vs. 87% in this study population) [58]. This highlights the need for primary care physicians to address sleep as a significant comorbidity for this population of children.

Comparing to reported data for clinical samples of typically developing children [32,59], the proportion of children who had a clinically significant sleep problem was much higher in our cohort of children with DS. This is consistent with the recent findings of Halstead et al., who similarly compared their cohort of children with neurodevelopmental disorders (including those with DS) to existing published data from typically developing children and found a much higher prevalence in the children with neurodevelopmental disorders [60].

Key findings of this study include the high prevalence of refractory SDB and, as anticipated, an equally high prevalence of non-respiratory, behavioural sleep problems. This highlights the need for sleep evaluation to not be focused solely on breathing issues. This is important as these additional issues add extra challenges for families, which may require alternative strategies for management.

Contrary to our hypothesis, this study did not show a relationship between either non-respiratory sleep problems or SDB with behaviour, function, or cognition in children with DS. This may reflect limitations of the measures used in this study, that in this population ongoing problems with daytime function are largely not driven by problems with the child’s sleep or that a temporal snapshot does not effectively evaluate the impact of past disease/treatments.

## 5. Conclusions

Children with Down syndrome referred to a sleep clinic were found to have a high rate of sleep disordered breathing even after initial treatment. This study highlights that an even higher proportion of those referred for snoring have non-respiratory sleep problems which could benefit from intervention. Further work is needed to demonstrate the potential benefits that improving sleep (both respiratory and non-respiratory) may have on the daytime function of young people with DS.

## Figures and Tables

**Table 1:** Demographic information and summary of surgical treatment prior to recruitment.

	Number of Participants (*n*)	Percentage (%)
Male/Female	20/22	48/52
Respiratory Condition	17	40
Thyroid Disease	12	29
Cardiac Disease	23	55
Another Comorbidity	19	45
	Median	IQR
Age at Baseline Assessment (yrs)	7.0	(4.5, 9.9)
BMI Percentile	50th	(25, 75)
Absolute BMI (Kg/m^2^)	17.4	(16.4, 21.6)
	Number of Participants (*n*)	Percentage %
Proportion of Children with BMI ≤2nd Percentile	2	5
Proportion of Children with BMI ≥90th Percentile	6	14
Sum	8	19
Surgical Treatment Prior to Recruitment		
Adenotonsillectomy	24	57.14
No Surgery	10	23.81
Adenoidectomy only	6	14.29
Tonsillectomy only	2	4.76
Sum	42	100.00

**Table 2:** Sleep data (CSHQ *n* = 42 and PSG *n* = 36) for participants.

CSHQ Total Score	Percentage (%)
Proportion of Children clinically elevated total score >41	86 (*n* = 36)
CSHQ Total Score	52.5 (46.5, 62.5)
CSHQ Sub-scores (max possible score)	Median (IQR)
Bedtime Resistance (35)	16.5 (12.0, 20.3)
Sleep Onset Delay (5)	2.0 (2.0, 3.0)
Sleep Anxiety (10)	5.0 (2.0, 6.0)
Night Wakings (15)	7.0 (5.8, 9.2)
Parasomnias (15)	7.5 (6.0, 9.0)
Sleep Disordered Breathing (5)	3.0 (2.8, 4.0)
Daytime Sleepiness (20)	9.0 (7.8, 11.0)
Questions with >20% Respondents scoring >3	Percentage Respondents (%)
Q3: Child falls asleep alone in own bed	45.2
Q7: Child needs parent in the room to fall asleep	45.2
Q8: Child resists going to bed at bedtime	21.5
Q11: Child is restless and moves a lot during sleep	76.2
Q12: Child moves to someone else’s bed during the night	23.8
Q14: Child snores loudly	45.2
Q16: Child naps during the day	28.5
Q17: Child wakes up once during the night	23.8
Q20: Child wakes up very early in the morning	40.4
Q21: Child falls asleep while involved in activities	47.6
OSA on PSG	
Proportion of children with total OAHI ≥ 1/h	61% (n = 22)
Proportion of children with total OAHI > 2/h	44% (n = 16)
Proportion of children with No OSA (OAHI < 1/h)	39% (n = 14)
Proportion of children with Mild OSA (OAHI (1–5/h)	28%(n = 10)
Proportion of children with Moderate OSA (OAHI 5–10/h)	14% (n = 5)
Proportion of children with Severe OSA (OAHI > 10/h)	19% (n = 7)
PSG Parameter	Median (IQR)
PSG Sleep Efficiency (%)	89 (80.5, 94)
PSG Total AHI (per h)	5.2 (1.90, 11.1)
PSG Total OAHI (per h)	1.6 (0.4, 6.7)
PSG REM OAHI (per h)	3.2 (1.3, 17.4)
PSG Total CAI (per h)	1.9 (0.7, 3.5)
PSG REM CAI (per h)	2.9 (1.0, 6.6)
PSG CO_2_ Maximum (mmHg)	50.7 (48.6, 56.9)
PSG Total Arousal Index (per h)	8.05 (5.5, 10.1)

**Table 3:** Life Habits (Life-H) Questionnaire data for participants (*n* = 40).

Measure	DS_study_ (3–16 yrs) Median (IQR) *n* = 40
Life H Total Score	4.4 (3.6, 5.6)
Life H Nutrition Score	4.2 (2.3, 5.6)
Life H Fitness Score	6.7 (5.6, 8.5)
Life H Personal Score	3.3 (1.9, 5.0)
Life H Communication Score	2.9 (2.0, 4.4)
Life H Housing Score	7.0 (5.2, 8.7)
Life H Mobility Score	4.4 (2.9, 6.4)
Life H Responsibilities Score	3.1 (1.4, 4.6)
Life H Relationships Score	8.3 (6.7, 10.0)
Life H Community Score	3.3 (0, 3.7)
Life H Education Score	3.3 (2.1, 5.4)
Life H Recreation Score	3.6 (2.8, 4.9)

**Table 4:** Child Behaviour Checklist (CBCL) data for participants (*n* = 42).

Measure	Median (IQR)	Participants in Clinical Concern/ Borderline Clinical Concern Range (%)
Total T-Score	57 (51.8, 64)	35.7
Internalising Problems T-Score	54.5 (49.8, 61)	31
Externalising Problems T-Score	55.5 (46.8, 61.3)	33.3
Anxious/Depressed T-Score	51 (50, 53)	7.1
Withdrawn/Depressed T-Score	59.5 (53.8, 64.5)	19
Somatic Complaints T-Score	59.5 (55.3, 65.3)	21.4
Social Problems T-Score	62 (54, 69)	21.4
Thought Problems T-Score	62 (54, 70)	23.8
Attention Problems T-Score	60 (55, 68.3)	31
Rule Breaking Behaviour T-Score	56 (51, 63)	9.5
Aggressive Behaviour T-Score	54 (50, 61)	16.7

**Table 5:** Stanford Binet-5 (SB-5) cognitive data for participants (*n* = 30).

Measure	Median (IQR)	No. Participants within Normal Range (Score ≥70)	No. Participants with Mild Impairment (Score 55–69)	No. Participants with Moderate Impairment (Score 40–54)	No. Participants with Severe Impairment (Score <40)
SB-5 Non-Verbal IQ Score	52.5 (47.0, 69.8)	7 (23%)	7 (23%)	16 (53%)	0
SB-5 Verbal IQ Score	50.0 (43.0, 58.3)	1 (3%)	8 (27%)	21 (70%)	0
SB-5 Full Scale IQ Score	50.0 (43.0, 62.0)	3 (10%)	8 (27%)	19 (63%)	0
SB-5 Fluid Reasoning Score	54.5 (47.0, 65.8)	6 (20%)	9 (30%)	15 (50%)	0
SB-5 Knowledge Score	58.5 (55.0, 77.8)	10 (33%)	14 (47%)	6 (20%)	0
SB-5 Quantitative Reasoning Score	56.0 (50.0, 77.75)	4 (13%)	13 (43%)	13 (43%)	0
SB-5 Visual-Spatial Processing Score	56.0 (48.0, 62.0)	3 (10%)	13 (43%)	14 (47%)	0
SB-5 Working Memory Score	54.0 (48.0, 60.8)	5 (17%)	9 (30%)	16 (53%)	0

## Data Availability

Data available on request due to restrictions. The data presented in this study are available on request from the corresponding author. The data are not publicly available due to participant privacy.
